# Septic arthritis as a severe complication of elective arthroscopy:clinical management strategies

**DOI:** 10.1186/1754-9493-3-6

**Published:** 2009-03-31

**Authors:** Chlodwig Kirchhoff, Volker Braunstein, Jochen Paul, Andreas B Imhoff, Stefan Hinterwimmer

**Affiliations:** 1 Department of Orthopedic Sports Surgery, Klinikum Rechts der Isar, Technische Universitaet, Ismaningerstrasse 22, D-81675 Munich, Germany; 2 Department of Traumatology and Orthopedic Surgery – Campus Innenstadt, Ludwig-Maximilians Universitaet, Nussbaumstrasse 20, D-80336 Munich, Germany; 3 AO Research Institute, Clavadelerstrasse 8, CH-7270, Davos, Switzerland

## Abstract

Infection of a peripheral joint following arthroscopic surgery presents with an incidence of approximately 0.42% an extremely rare entity. However, septic arthritis is a serious situation possibly leading to an irreparable joint damage. Especially at delayed diagnosis patients' safety can be endangered severely. Only few precise statements regarding diagnosis and therapy have been published so far. Besides an accurate analysis of the patient's anamnesis and the assessment of the C-reactive protein especially arthrocentesis is required for diagnostic workup. For early stage infections arthroscopic therapy is proven to be of value. In addition a calculated and consecutive germ-adjusted antibiotic therapy is essential. In case of persisting signs of infection the indication for re-arthroscopy or conversion to open revision has to be stated in time. The number of necessary revisions is dependent on the initial stage of infection. For pain therapy postoperative immobilization of the affected joint is occasionally essential, if otherwise possibly early mobilization of the joint should be performed.

## Introduction

Although septic arthritis following arthroscopic surgery is a very rare incident it is a crucial issue, significantly endangering patient's safety [1,2]. In case of delayed diagnosis and therapy infection causes severe impairment of the affected joint and even lead to a life threatening situation [3]. On the one hand there are certain patient-specific risk factors such as immunosuppressive diseases [4,5]. On the other hand arthroscopy specific factors such as sterilization of the increasingly complex equipment, duration of surgery and the extent of arthroscopic intervention appear to influence the risk of joint infections [6]. Based on the current literature the present article discusses the relevant recommendations regarding prevention, diagnosis and therapy of septic arthritis and is thus intended to be a guideline for the orthopedic surgeon.

### Epidemiology

Major reasons for septic arthritis are haematogenous pathogen spread, intraarticular injections, penetrating injuries and open-surgeries [7]. In contrast arthroscopic intervention plays only a subsidiary role. Jerosch et al. reported infection rates of up to 0.42% following arthroscopy [2]. Recently the German institute for ambulatory arthroscopy (BVASK) reported an incidence rate of 0.13% following ambulatory arthroscopy [8]. They evaluated 51.079 surgeries in 66 centers between 2001 and 2008. They furthermore stated, that depending on the duration of the surgery, the number of previous interventions, the extent of the intraoperative procedure as well as previous steroid injections the infection rate seems to increase [8]. In this context several authors additionally found combinations of arthroscopic and open surgery techniques to increase the risk for septic arthritis [6,9].

Different joints reveal specific incidence rates. The lowest of approximately 0.8% is found for the elbow, followed by the knee with 1% and the shoulder with up to 3.4% [10]. Regarding hip arthroscopy Mc Carthy et al. reported not a single case of septic arthritis in 1500 patients [11]. The ankle joint appears to have the highest incidence of septic arthritis of up to 5.7% [2,12].

### Pathogenesis

Initially, bacteria are conveyed via arthroscopy medium or instruments into the joint or the periarticular tissue and deposit in the synovial membrane leading to an acute inflammatory response. As the synovial does not exhibit a limiting barrier bacteria easily infiltrate the synovial fluid and cause purulent infection [2]. Therefore the main problem is the imminent danger of infection dispersion throughout the complete joint. Furthermore there might be protracted synovitis as well as irreversible cartilage damage.

### Risk Factors

The immune competent organism is capable to eliminate pathogens via phagocytosis by synovial cells [13]. Only very few patients who intraoperatively had been exposed to pathogens consecutively develop wound infection and joint infection respectively. Thus, the majority of postoperative infections might be determined by endogenous factors. Therefore the surgeon can influence only a limited extent [14]. Not-adjusted diabetes mellitus, liver cirrhosis, dialysis, rheumatoid arthritis and malignant diseases are potential predisposing factors [15]. In contrast, several studies regarding the impact of human immunodeficiency virus (HIV) infection on the onset of septic arthritis yielded only marginal influence [4]. Besides systemic reasons, local factors such as osteoarthritis have to be mentioned (see table 1). Another relevant risk factor is any precedent systemic steroid or immunosuppressive therapy. An eminently high risk is retrieved by intraarticular steroid application [16].

**Table 1:** Patient specific risk factors

Malignant underlying diseases
Rheumatoid arthritis
Immunodeficiency
Renal dialysis obligation
Liver cirrhosis
Nicotine abuse
Obesity
Not-adjusted diabetes mellitus
High age
Medicamentous immunosuppression

### Prevention

#### Asepsis/Preparation

On the one hand recognition of patient's specific risk factors and adaption of therapy is crucial [17], on the other hand exogenous risk factors have to be minimized. Besides intraoperative asepsis attention should particular focus on the preparation and sterilization of endoscopic instruments, needles, optics and electronic components [18].

#### Perioperative Antibiotic Prophylaxis (PAP)

Data in the current literature do not support a general recommendation on PAP [19]. A retrospective review on more than 300 arthroscopic knee interventions reported an infection rate of 0.15% in patients treated with PAP and of 0.16% in patients without PAP [19]. In contrast, there are several studies reporting adverse effects of a PAP including allergic reaction or Clostridium difficile associated diarrhea. Therefore the orthopedic surgeon has to identify the patient as well as the intervention specific risk factors requiring a PAP even for aseptic interventions [18]. PAP antibiotics should cover the relevant pathogens and be well tolerated. For arthroscopic surgery Cephalosporines of the 1^st ^or 2^nd ^generation are suitable due to their effectiveness against Staphylococcus Aureus [20]. Cephalosporines of the 3^rd ^and 4^th ^generation as well as Chinolones are not indicated for a routine PAP because of the chance of developing resistance. Furthermore these antibiotics are associated with a higher risk of Clostridium difficile infection [20]. In case of allergy towards Cephalosporines and/or incompatibility Clindamycin can be used. Vancomycin is indicated only in terms of second-line antibiotics and in case of Cephalosporine intolerance or Methicillin Resistant Staphylococcus Aureus (MRSA) colonization or infection [19].

Pharmacodynamically the best time point for antibiotics application is at 30 minutes before skin incision, therefore during onset of general anesthesia. A lot of studies have proven that repeated application compared to single application (single shot) of antibiotics does not offer a higher antibiotic coverage, but is associated with a higher incidence of adverse side effects [19]. Though, in surgery lasting more than 3–4 hours a second antibiotic application might be required [19].

### Diagnosis

Discrimination between physiological wound healing, postoperative irritation and joint infection is difficult. Several factors impede accurate diagnosis, i.e. in case of early functional aftercare, or initial stages of infection. Particularly degenerative damaged joints offer diagnostic difficulties to differentiate whether acute exacerbation of the pre-existing joint disease or a delayed infection after arthroscopy is present [21].

#### Anamnesis, Clinical Facts

Regarding anamnesis it is essential to accurately document previous medical and surgical interventions (i.e. the number of steroid injections) [2]. Although several authors claim classic symptoms of infection (rubor, dolor, calor, tumor and functio laesa) for the clinical diagnosis of septic arthritis their diagnostic value is only limited [22]. Following a meta-analysis of Margaretten et al. pain-related limited range of motion was present in 85%, swelling of a joint in 78% of the cases but only in 30% of the cases significant rubor or swelling was noted [15]. Moreover, these symptoms can also be present in cases of aseptic arthritis as well. Differentiation of joint infection to chondrocalcinosis, reactive arthritis (e.g. Lyme's disease) or Sudeck's syndrome can be challenging in some cases [22].

In cases of postoperative infection redness is present in the area of the surgical approach; pus can leak from the wound as well. During antibiotic or immunosuppressive therapy or in cohesion with immune-compromising diseases clinical symptoms might not be evident until advanced stages of septic arthritis. Besides local findings general symptoms can occur. Fever, chills, circulatory depression and acute shock pathology are some sorts of possibly evolving symptoms.

#### Laboratory Parameters

Mehta et al. demonstrated that analysis of white blood cell count (WBC) is only of limited diagnostic value [10]. He reported that almost 40% of patients presenting with acute septic arthritis had a leukocyte account of <10.000/ml. In contrast C-reactive protein (CRP) in these patients was significantly increased in more than 95% [10].

#### Diagnostic Arthrocentesis

If septic arthritis is suspected diagnostic arthrocentesis should be performed. In case of difficult approachable joints ultrasound guidance might help before a calculated antibiotic therapy is initiated. The macroscopic inspection of the aspirate may already indicate a possible infection [23]. Typically a viscous, purulent aspirate is extracted in bacterial joint infections. A Gram stain can be performed, whereas the microbiological analysis is obligatory for the confirmation of diagnosis and as guideline for the resistance-adapted antibiotic therapy. The leucocytes' account in these joint effusions is usually >50.000 leucocytes/mm^3 ^with more than 90% of the cells being polymorphonuclear cells. In this context it needs explicitly to be pointed out that low leukocyte numbers below 1000 – 10.000 leukocytes/mm^3 ^do not exclude joint infection. A cell count over 50.000/mm^3 ^in the joint aspirate is yet evidential for septic arthritis [2].

#### Imaging

**Figure 1:**
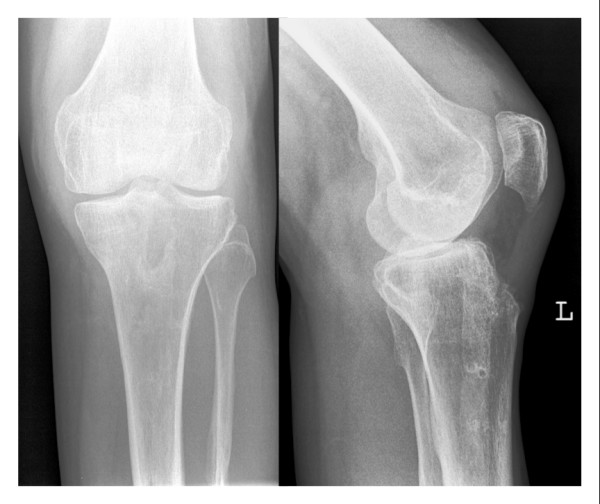
**37 year old female, 5 weeks following removal of a tibia nail, developed postoperative pain, swelling, warming and redness of knee joints**. Left knee joint: narrowing of joint space, subchondral osteolysis, Gaechter III (intraoperatively classified).

Although radiographs provide only important clues in the late phase of disease, they are mandatory for documentation (figure 1). Patients with suspicion for concomitant septic spread should undergo a CT-exam. MRI may be helpful in cases of chronic infections as well as septic spread [6,15]. It enables evaluation of extra-articular expansion in patients with a fulminant progression or distinct clinical signs [2,22].

### Therapy

One representative clinical study showed the distinct correlation between early diagnosis, therapy and clinical outcome [22]. Even if joint infection is only suspected surgical revision is indicated. The cornerstones of therapy are built by mechanical purification, release of pressure, antibiotic therapy and functional healing [24]. According to the recent literature procedures such as straight needle aspiration for joint-drainage or the flush-suck-treatment do not have therapeutic value any more [2,25].

#### Arthroscopic Surgery

**Figure 2:**
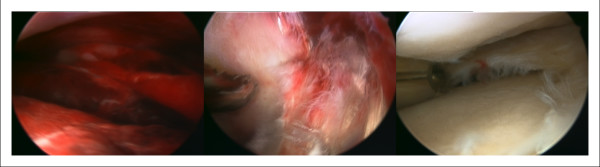
**48 year old male, ACL reconstruction 5 weeks ago, postoperative swelling, redness and hyperthermia, CRP 1.3 mg/dl**. Aspirate: Staph. epidermidis ++, Intra-OP Gaechter stage II

The complication rate and the number of exits during treatment of septic joint infections were evidently reduced via arthroscopic management. Regarding arthroscopic therapy an accepted stage-oriented procedure exists. The therapeutic management is usually determined by arthroscopic diagnosis and is primarily based on the classification of Gaechter [24] (see table 2). For most pediatric and adult cases of septic arthritis Gaechter stage I and II arthroscopic treatment according to intraoperative findings is adequate [24,26]. In arthroscopic procedure each joint compartment needs to be inspected, lavaged and released from necrotic tissue. A biopsy for microbiological and histological examination needs to be obtained during the surgery. Necrotic changes and adhesions in the area of the synovial membrane have to be removed compulsory. A primary synovectomy is not indicated for early stages of infection because of the physiological barrier through the synovial membrane. In cases of Gaechter stage III an intensive synovectomy along with necrosectomy, adhesiolysis and cartilage debridement should be performed if necessary (figure 2) [2]. Essential for lavage of the joint is the use of at least 10 to 15 liters of lavage fluid. The postoperative redon-drainages are removed after 24 to 48 hours and the tips of the drainages are sent in for microbiological analysis [24]. Consecutively a small meshed clinical observation and CRP-control is performed. Whenever the CRP does not fall or even tends to increase within 2 days after the first revision and simultaneous start of the antibiotic therapy a contemporary follow-up intervention is indicated [2].

**Table 2:** Arthroscopic staging; Gaechter [24]

**Stage I**
- turbis synovial
- redness of synovial membrane
- petechial bleeding
- no radiographic changes

**Stage II**
- severe inflammation with fibrin clots
- pus
- no radiographic changes

**Stage III**
- swelling of synovial membrane
- formation of compartments
- no radiographic changes

**Stage IV**
- Pannus with infiltration of cartilage
- subchondral osteolysis
- bony erosions and cysts

Most authors pronounce against an intraarticular therapy including anti-septic additives due to hereby possibly creating irreversible cartilage damages. Other treatment options include the outlay of the joint space with antibiotic straps or the intraarticular application of antibiotics (e.g. Gentamycin). These methods do not seem justifiable in cases of intact articular cartilage because these methods might lead to mechanical damage during the postoperative mobilization. Different clinical examinations have shown that an adequate high synovial antibiotics level is reached following intravenous application. Moreover Argen et al. described the appearance of synovitis after intraarticular instillation of antibiotics [27].

#### Open Surgery

Despite all advantages of arthroscopic therapy the limits in case of joint infection should be kept in mind. Thus a straight arthroscopic approach is not advisable if osseous affection of the infection is suspected. An arthroscopic therapy management following primary open surgery is not useful when the infection focus is suspected in the area of open surgical approach (i.e. extra-articular localization) [2].

#### Antibiotic Therapy

In septic arthritis a positive pathogen proof is not found regularly and there is a broad range between 60% and 100% of proven pathogens as reported in literature. The microbiological pathogen proof is especially aggravated if the patient had already been treated antibiotically before the extraction of assay material was performed. Previous to the presentation of the antibiogram the peri- and postoperative antibiotic coverage is performed using a staphylococcus-potent antibiotic [22]. Although in the current literature no general agreement exists regarding the initial parenteral antibiotic therapy and the consecutive oral antibiosis a focused antibiotics therapy should be performed after the antibiogram for at least 4–6 weeks after the CRP-level was normalized [22]. There is an increasing prevalence of MRSA with has to be considered particularly [28]. To optimize antibiotic therapy an interdisciplinary approach in cooperation with a microbiological department plays an increasingly important role for the orthopedic surgeons.

### Aftercare

Postoperative immobilization of the affected joint can occasionally be necessary for pain treatment. On the first postoperative day physiotherapeutic treatment with support of movement splints (continuous passive motion, CPM) and cryotherapy is indicated. The CPM splint prevents adhesions, improves local cartilage nutrition, evacuates lysosomal enzymes and purulent exsudation and stimulates chondrocytic matrix synthesis [2]. The postoperative partial weight bearing of the joint should be guided by local clinical findings, subjective complaints of the patient and intraarticular documented damage. Full weight bearing is allowed 6 weeks post surgery at the earliest even for patients without complications postoperative to preserve busted hyaline articular cartilage [22].

### Prognosis

Prognosis of infectious arthritis distinctly depends on the in-time diagnosis and the initiation of adequate therapy within the first couple of days after incidence of symptoms. Also life-threatening stages can develop consecutively. Especially in cases of pre-existing chronic poly-arthritis prognosis of septic arthritis is significantly worse. The mortality rate ranges at approximately 20% for patients with chronic polyarthritis compared to 5–10% for other groups [29]. The respective prognosis of the affected joint depends in particular on the primary and secondary damage to the intra-articular structure.

## Key messages

• Prognosis depends on the in time diagnosis and therapy within the first couple of days after the incidence of infection symptoms.

• When suspecting septic arthritis a diagnostic arthrocentesis should be performed before the beginning of an (calculated) antibiotic treatment, in difficult cases performed via ultrasound-guidance.

• The operative infection management is determined by following arthroscopic diagnosis assessment as described by Gaechter.

• Postoperatively immobilization of the affected joint can be necessary regarding the patients' pain or otherwise physiotherapy should be performed.

• During early stages of infection daily re-evaluation is necessary regarding possibly appearing pain or other clinical signs of infection in order to recognize a septic spread as soon as possible.

## Abbreviations

BVASK: German Institution For Ambulatory Arthroscopy; CPM: Continous Passive Motion; CRP: C-reactive Protein; CT: Computed Tomography; HIV: Human Immunodeficiency Virus; MRI: Magnet Resonance Imaging; MRSA: Methicillin Resistant Staphylococcus Aureus; PAP: Perioperative Antibiotic Prophylaxis; WBC: White Blood Cell Count.

## Competing interests

The authors declare that they have no competing interests.

## Authors' contributions

CK and VB contributed to review design, literature analysis and drafted the manuscript. JP, ABI and SH contributed to manuscript review. All authors read and approved the final manuscript.
